# Association of physical activity with arterial stiffness: the mediating role of the visceral fat metabolism score

**DOI:** 10.20945/2359-4292-2026-0094

**Published:** 2026-08-12

**Authors:** Huihua Jiang, Xiaoli Shao, Liqin Jiang, Teng Zhang

**Affiliations:** 1 Department of Neurology, Chun’an County First People’s Hospital, 311700 Hangzhou, Zhejiang, China

**Keywords:** Physical activity, arterial stiffness, metabolic score for visceral fat, estimated pulse wave velocity

## Abstract

**Objective:**

We investigated the association between physical activity (PA) and arterial stiffness (AS) in Chinese middle-aged and older adults, systematically evaluating the potential mediating role of the Metabolic Score For Visceral Fat.

**Subjects and methods:**

Data were drawn from the 2015 China Health and Retirement Longitudinal Study. PA was assessed using the International Physical Activity Questionnaire, and AS was determined by estimated pulse wave velocity. Multivariable logistic regression, restricted cubic spline models, and Bootstrap mediation analysis were applied for statistical analyses.

**Results:**

In the fully adjusted model, each standard deviation increase in PA intensity was associated with a 22% reduction in AS risk (odds ratio [OR] = 0.78, 95% confidence interval [CI]: 0.72–0.84, *p* < 0.001). Compared to low-intensity PA, both moderate-intensity (OR = 0.75, 95% CI: 0.61–0.92, *p* = 0.005) and high-intensity PA (OR = 0.57, 95% CI: 0.48–0.68, *p* < 0.001) were significantly associated with a lower risk of AS. Restricted cubic spline analysis revealed a nonlinear association, with AS risk decreasing as PA intensity increased (*p*-nonlinear = 0.034). Mediation analysis indicated that Metabolic Score For Visceral Fat significantly mediated the negative correlation between PA and AS, accounting for 11.4% of the total effect.

**Conclusion:**

Among middle-aged and older Chinese adults, higher levels of PA are associated with a lower risk of AS, with this association partially mediated by improvements in visceral fat metabolism.

## INTRODUCTION

Globally, cardiovascular disease (CVD) accounts for a substantial proportion of deaths and disabilities. Effective primary prevention of CVD depends on the early detection and management of vascular abnormalities during the subclinical phase (^1^,^2^). Among the various early markers of vascular function, arterial stiffness (AS), a core manifestation of vascular aging, is a well-established independent predictor of incident CVD and all-cause mortality (^3^–^5^). Therefore, developing accurate and convenient tools to assess AS is key for risk stratification in large populations. Currently, carotid-femoral pulse wave velocity is considered the “gold standard” for evaluating AS. However, the complex method involved hinders its widespread use in population-based research (^6^). To address this challenge, estimated pulse wave velocity (ePWV), calculated from routine parameters such as age and blood pressure, has been proposed. It is a proven independent predictor of mortality from all causes and specifically from CVD in the general population, making it an ideal alternative for assessing vascular health in large cohorts (^7^).

Routine physical activity (PA) is established as an effective non-pharmacological intervention for improving and slowing arterial aging as part of a vascular-healthy lifestyle (^8^). This effect has been validated across diverse populations; for instance, sustained aerobic exercise has been reported to significantly reduce the degree of AS in middle-aged and older women (^9^). Among patients with metabolic syndrome, moderate-to-vigorous PA has been associated with marked improvements in AS parameters (^10^). Notably, visceral fat (VF) is likely central to the beneficial effects of PA on arterial AS (^11^). Evidence suggests that higher PA levels are strongly associated with lower VF content (^12^), and accumulation of VF is closely linked with an increased risk of AS (^13^). Therefore, reductions in VF likely mediate the improvement in AS observed with increased PA.

Despite growing evidence for multiple pathways, the specific pathways by which PA modulates AS warrant further investigation (^14^). As a critical nexus between metabolic dysregulation and vascular aging, the role of VF in the relationship between PA and AS has not been systematically validated in large populations (^15^). Utilizing data from the China Health and Retirement Longitudinal Study (CHARLS), this study aimed to investigate the association between PA and AS in middle-aged and older Chinese adults, by analyzing the interconnected roles of PA, the Metabolic Score for Visceral Fat (METS-VF), and ePWV. Specifically, we sought to determine whether VF mediates the association between PA and AS, thereby providing novel epidemiological evidence elucidating the relationship between PA and vascular health.

## SUBJECTS AND METHODS

### Study design

This study used data from the 2015 CHARLS survey, a nationally representative longitudinal survey conducted in China. CHARLS systematically collects multidimensional information on health status, socioeconomic characteristics, and other factors among Chinese individuals aged ≥45 years. The survey adopted a multistage, stratified probability sampling method, covering 28 provinces (including autonomous regions and municipalities) across China. Its rigorous method and high-quality data collection are well established (^16^). Notably, all data collection protocols for the CHARLS database were reviewed and approved by the Biomedical Ethics Committee of Peking University. As written informed consent was secured from all participants at enrollment, a separate ethical review for this analysis was not required.

**Figure 1.**
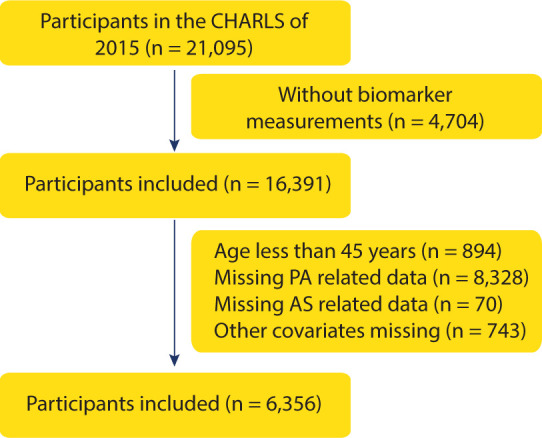
Inclusion and exclusion criteria.

The initial sample comprised 21,095 participants. An iterative filtering process was applied based on predefined criteria: (1) exclusion of individuals who did not participate in the 2015 physical examination (n = 4,704); (2) exclusion of those aged <45 years (n = 894); (3) exclusion of those with missing data on PA-related variables (n = 8,328); (4) exclusion of those with missing data for AS assessment indicators (n = 70); and (5) exclusion of those with incomplete covariate data (n = 743). Ultimately, 6,356 participants were included in the final analysis. The participant selection flowchart is presented in **Figure 1**.

### Definition and measurement of variables

#### Exposure variable

Physical activity level was defined as the exposure variable, assessed using the internationally recognized metabolic equivalent of task (MET) system and strictly following the standardized protocol of the International Physical Activity Questionnaire (^17^). Based on questionnaire responses regarding activity duration, daily PA time was categorized into five levels (0 min, 10–29 min, 30–119 min, 120–239 min, and ≥240 min), with midpoint values used for quantitative analysis. Activity types were classified into three distinct intensity grades: vigorous-intensity PA (assigned a MET value of 8.0), including strenuous activities such as climbing, running, and heavy farming; moderate-intensity PA (MET value of 4.0), including brisk walking and Tai Chi; and low-intensity PA (MET value of 3.3), including light activities such as leisurely walking. Total weekly PA was calculated as MET value × number of activity days per week × daily activity duration (minutes). Following International Physical Activity Questionnaire criteria, intensity was categorized as low (<600 MET-min/week), moderate (600–3000 MET-min/week), or high (>3000 MET-min/week). To ensure data quality, outliers were identified using the interquartile range (IQR) method. Values falling below Q1 − 1.5 × IQR or above Q3 + 1.5 × IQR were treated as missing to maintain the reliability of the study findings.

#### Mediating variable

The METS-VF, a composite indicator of visceral adiposity derived from metabolic profiles, was used as the mediating variable (^18^,^19^). This scoring system comprises two core components: the metabolic score for insulin resistance (METS-IR) and the waist-to-height ratio (WHtR). METS-IR was calculated as [ln(2 × fasting glucose + triglycerides) × body mass index (BMI)] / ln(high-density lipoprotein cholesterol), effectively assessing the metabolic activity of adipose tissue using blood glucose, lipid, and body weight. The formula for WHtR is waist circumference/height, a morphological indicator that accurately characterizes the fat distribution features of central obesity. METS-VF was then computed using a validated multiple regression equation: 4.466 + 0.011 × (ln(METS-IR))^3^ + 3.239 × (ln(WHtR))^3^ + 0.319 × sex + 0.594 × (ln(age)). All laboratory indicators were measured with standardized methods: fasting glucose and triglycerides in mg/dL; high-density lipoprotein cholesterol in mg/dL; BMI in kg/m^2^; waist circumference and height in cm; and sex coded as a binary variable (male = 1, female = 0). By integrating metabolic, morphological, and demographic information, this scoring system provides a more robust assessment of the pathological burden imposed by VF.

#### Outcome variable

Arterial stiffness was the outcome variable, measured using the widely validated, noninvasive ePWV calculated from age and blood pressure. ePWV was derived using a published regression formula: 9.587 − 0.402 × age + 4.560 × 10^-3^ × age^2^ − 2.621 × 10^-5^ × age^2^ × mean blood pressure + 3.176 × 10^-3^ × age × mean blood pressure − 1.832 × 10^-2^ × mean blood pressure, where mean blood pressure was defined as diastolic blood pressure + 0.4 × (systolic blood pressure - diastolic blood pressure). Consistent with current consensus in cardiovascular research, AS was defined as ePWV ≥10 m/s (^20^,^21^). The cutoff value was based on the following evidence: (1) validation studies have demonstrated that ePWV ≥10 m/s exhibits good consistency and diagnostic accuracy compared with the gold standard carotid-femoral artery pulse wave velocity (≥10 m/s) (^22^); (2) large prospective cohort studies have verified that ePWV ≥10 m/s is significantly associated with increased risk of cardiovascular and cerebrovascular events, such as myocardial infarction and stroke (^23^); and (3) several recent studies utilizing representative databases, including NHANES and CHARLS (^24^–^26^), have adopted ePWV ≥10 m/s as the operational definition of AS to enhance the comparability of findings across populations.

#### Covariates

Based on previous evidence, we accounted for potential confounders that could affect the PA-AS association across multiple domains. Demographic characteristics included age, sex (male/female), level of education (illiterate/primary school or below/secondary school or above), marital status (with or without partner), and residential area (rural/urban) (^27^). Lifestyle factors included current smoking status (yes/no) and current alcohol drinking (yes/no). Health status-related variables included BMI [categorized as normal ( <24 kg/m^2^), overweight (24–28 kg/m^2^), and obese (≥28 kg/m^2^)], and three common chronic diseases: diabetes, dyslipidemia, and CVD (^28^). Diagnostic criteria were as follows: Diabetes was defined based on any of the following: (1) fasting plasma glucose ≥126 mg/dL; (2) glycosylated hemoglobin ≥6.5%; or (3) self-reported physician diagnosis. Dyslipidemia was defined by any of the following: (1) total triglycerides ≥150 mg/dL; (2) total cholesterol ≥240 mg/dL; (3) high-density lipoprotein cholesterol <40 mg/dL; (4) low-density lipoprotein cholesterol ≥160 mg/dL; or (5) self-reported physician diagnosis. These cutoff values were referenced from several previous studies using the CHARLS database (^29^–^31^) and have been widely applied in large epidemiological cohorts to identify individuals with clinically significant dyslipidemia, including within CHARLS. Cardiovascular disease was assessed via standardized questions: participants were classified as having CVD if they reported a physician diagnosis of heart attack, coronary heart disease, angina, congestive heart failure, or stroke.

### Statistical analysis

All statistical analyses were performed using R software (v. 4.4.2). Baseline characteristics were summarized by AS status. Categorical variables are presented as n (%), and continuous variables as mean. Comparisons between groups were conducted using analysis of variance for continuous variables and the chi-square test for categorical variables, with the *tableone* package used to generate the baseline table. PA was stratified into three levels based on intensity: low, moderate, and high. A multivariable logistic regression model was employed to assess the association between different PA levels and AS. Model 1 was unadjusted. Model 2 adjusted for age, sex, level of education, marital status, and residential area. Model 3 further adjusted for smoking, alcohol consumption, BMI, diabetes, dyslipidemia, and CVD in addition to variables in Model 2. All logistic regression analyses used the *stats* package, with the low-intensity group serving as the reference. To explore the heterogeneity in the PA-AS association across population subgroups, we performed subgroup analyses stratified by age and sex, and tested for interaction effects. All subgroup and interaction analyses were conducted using the *jstable* package. Restricted cubic spline (RCS) models were used to examine potential nonlinear relationships between PA and AS, with RCS curves plotted using the *rms* package.

Potential mediating effects of VF-related indicators on the association between PA and AS were examined using the bootstrap method with 500 resamplings. We evaluated whether METS-VF and its core components (i.e., METS-IR and WHtR) mediated the association between PA and AS, and estimated the direct, indirect, and total effects separately. To mitigate multicollinearity, the mediating effects of METS-VF, METS-IR, and WHtR were each assessed in separate models, with only one mediator included per model. All models were fully adjusted for covariates including age, sex, level of education, marital status, residential area, smoking, alcohol consumption, diabetes, dyslipidemia, and CVD. The BMI was retained in all models to control for its confounding influence, enabling a more precise estimation of the independent mediating effect of METS-VF after accounting for general adiposity. Given the cross-sectional design, the reported mediation effects should be interpreted as statistical associations and considered exploratory evidence for potential mechanistic pathways. All mediation analyses were performed using the mediation package. Furthermore, considering that the applicability of defining AS as ePWV ≥10 m/s may vary by population, we conducted supplementary sensitivity analyses by defining AS as ePWV above the 75^th^ percentile to confirm the robustness of our primary findings. All statistical tests were two-sided, and *p* < *0*.05 was considered statistically significant.

## RESULTS

### Baseline characteristics

This study included 6,356 participants with a mean age of 61.0 ± 9.4 years, among whom 2,504 (39.4%) were diagnosed with AS. Compared with their non-AS counterparts, participants with AS were older and had a higher prevalence of male sex, single marital status, and current smoking, along with lower educational attainment (*p* < *0*.001). In terms of health status, the AS group also had a markedly higher prevalence of diabetes, dyslipidemia, and CVD (*p* < *0*.001). Additionally, both systolic and diastolic blood pressure levels were significantly elevated in the AS group. Regarding PA, the proportion of participants engaging in moderate- and high-intensity activities was significantly lower in the AS group (*p* < *0*.001), suggesting a substantial association between PA levels and AS status (**Table 1**).

**Table 1.** Baseline characteristics of the study participants

Variable	Total, n (%)	Arterial stiffness, mean (SD)		p-value
		Absent	Present	
n	6356	3852	2504	
Age (years)	61.0 (9.4)	55.8 (6.1)	69.0 (7.6)	<0.001
Sex (%)				<0.001
Female	3376 (53.1)	2179 (56.6)	1197 (47.8)	
Male	2980 (46.9)	1673 (43.4)	1307 (52.2)	
Level of education (%)				<0.001
Illiterate	1636 (25.7)	824 (21.4)	812 (32.4)	
Elementary school or below	2674 (42.1)	1532 (39.8)	1142 (45.6)	
Middle school or above	2046 (32.2)	1496 (38.8)	550 (22.0)	
Marital status (%)				<0.001
Single	778 (12.2)	258 (6.7)	520 (20.8)	
Married	5578 (87.8)	3594 (93.3)	1984 (79.2)	
Residential area (%)				0.886
Rural	3972 (62.5)	2404 (62.4)	1568 (62.6)	
Urban	2384 (37.5)	1448 (37.6)	936 (37.4)	
Smoking status (%)				<0.001
No	3586 (56.4)	2302 (59.8)	1284 (51.3)	
Yes	2770 (43.6)	1550 (40.2)	1220 (48.7)	
Alcohol consumption (%)				0.074
No	4164 (65.5)	2490 (64.6)	1674 (66.9)	
Yes	2192 (34.5)	1362 (35.4)	830 (33.1)	
Body mass index (kg/m^2^)	23.9 (3.8)	23.9 (3.6)	24.0 (4.1)	0.735
Diabetes (%)				<0.001
No	5325 (83.8)	3307 (85.9)	2018 (80.6)	
Yes	1031 (16.2)	545 (14.1)	486 (19.4)	
Dyslipidemia (%)				<0.001
No	3676 (57.8)	2313 (60.0)	1363 (54.4)	
Yes	2680 (42.2)	1539 (40.0)	1141 (45.6)	
Cardiovascular disease (%)				<0.001
No	5235 (82.4)	3328 (86.4)	1907 (76.2)	
Yes	1121 (17.6)	524 (13.6)	597 (23.8)	
Systolic blood pressure (mm/Hg)	127.4 (20.0)	118.3 (14.2)	141.5 (19.7)	<0.001
Diastolic blood pressure (mm/Hg)	75.0 (11.7)	71.9 (10.0)	79.7 (12.6)	<0.001
Physical activity				<0.001
Light	1074 (16.9)	516 (13.4)	558 (22.3)	
Moderate	1430 (22.5)	808 (21.0)	622 (24.8)	
Vigorous	3852 (60.6)	2528 (65.6)	1324 (52.9)	

SD: standard deviation.

### Association between PA and the risk of AS

Multivariable logistic regression analysis was conducted to assess the relationship between PA levels and AS risk, adjusting for potential confounders. Detailed results are shown in **Table 2**. The analysis revealed a significant negative correlation between PA and the risk of AS. In the fully adjusted Model 3, each standard deviation increase in PA intensity was associated with a 22% lower risk of AS (OR = 0.78, 95% CI: 0.72–0.84). Analysis by PA intensity level demonstrated a clear dose-response relationship. Compared with the low PA group, both moderate- and high-intensity PA were associated with a lower risk of AS. In the fully adjusted Model 3, moderate-intensity activity was linked to a 25% reduction in AS risk (OR = 0.75, 95% CI: 0.61–0.92), while high-intensity activity corresponded to a 43% decreased risk (OR = 0.57, 95% CI: 0.48–0.68). Sensitivity analyses yielded findings consistent with the primary analyses (**Supplementary Table S1**).

**Table 2.** Association between physical activity and arterial stiffness

Variable	Model 1		Model 2		Model 3	
	OR (95%CI)	*p*-value	OR (95%CI)	*p*-value	OR (95%CI)	p-value
Per-SD	0.74 (0.70–0.78)	<0.001	0.75 (0.70–0.80)	<0.001	0.78 (0.72–0.84)	<0.001
Categorical						
Light	1 (Reference)		1 (Reference)		1 (Reference)	
Moderate	0.71 (0.61–0.83)	<0.001	0.76 (0.62–0.93)	0.009	0.75 (0.61–0.92)	0.005
Vigorous	0.48 (0.42–0.56)	<0.001	0.55 (0.47–0.66)	<0.001	0.57 (0.48–0.68)	<0.001
*p* (trend)		<0.001		<0.001		<0.001

**Model 1** was unadjusted. **Model 2** was adjusted for age, sex, educational level, marital status, and residential area. **Model 3** was further adjusted for age, sex, educational level, marital status, residential area, smoking, alcohol consumption, body mass index, diabetes, dyslipidemia, and cardiovascular disease. OR: Odds ratio; CI: confidence interval.

### Dose-response association between PA and AS

**Figure 2.**
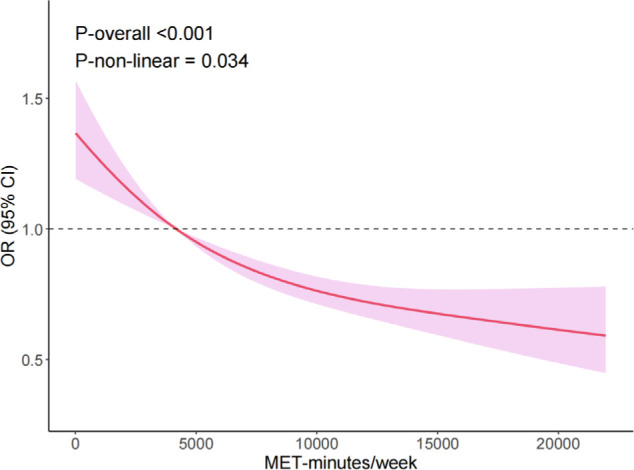
Dose-response association between physical activity and arterial stiffness.

We used RCS to investigate the potential nonlinear dose-response relationship between PA and AS risk. As shown in **Figure 2**, a nonlinear dose-response relationship was observed between PA and the risk of AS (*p* for overall association < 0.001, *p* for nonlinearity = 0.034).

### Subgroup analysis and interaction tests

To assess the consistency of the association between PA and AS across various populations, subgroup analyses were conducted, stratified by age, sex, and chronic disease status (**Table 3**). A markedly lower AS risk with high-intensity PA was observed across all subgroups, although no statistically significant interactions were detected (*p* > 0.05). These results suggest that the protective effect of PA against AS is robust and generally consistent across individuals with different ages, sexes, and chronic disease statuses.

**Table 3.** Subgroup analysis

Variable	n	Level	OR (95%CI)	*p*-value	*p*_(interaction)_
Age (years)					0.128
<60	2911	Light	Reference		
		Moderate	0.65(0.42–1.01)	0.055	
		Vigorous	0.68(0.47–0.97)	0.035	
≥60	3445	Light	Reference		
		Moderate	0.77(0.61–0.98)	0.031	
		Vigorous	0.54(0.45–0.67)	<0.001	
Sex					0.203
Female	3376	Light	Reference		
		Moderate	0.69(0.52–0.92)	0.011	
		Vigorous	0.49(0.39–0.63)	<0.001	
Male	2980	Light	Reference		
		Moderate	0.84(0.62–1.12)	0.230	
		Vigorous	0.67(0.52–0.86)	0.002	
Hypertension					0.146
No	3677	Light	Reference		
		Moderate	0.66(0.48–0.92)	0.012	
		Vigorous	0.51(0.39–0.67)	<0.001	
Yes	2679	Light	Reference		
		Moderate	0.96(0.70–1.31)	0.804	
		Vigorous	0.78(0.59–1.01)	0.054	
Diabetes					0.850
No	5325	Light	Reference		
		Moderate	0.77(0.61–0.96)	0.022	
		Vigorous	0.58(0.48–0.7)	<0.001	
Yes	1031	Light	Reference		
		Moderate	0.67(0.42–1.06)	0.086	
		Vigorous	0.54(0.36–0.80)	0.003	
Dyslipidemia					0.772
No	3676	Light	Reference		
		Moderate	0.72(0.54–0.95)	0.022	
		Vigorous	0.59(0.47–0.75)	<0.001	
Yes	2680	Light	Reference		
		Moderate	0.76(0.57–1.02)	0.072	
		Vigorous	0.54(0.42–0.7)	<0.001	
Cardiovascular disease					0.384
No	5235	Light	Reference		
		Moderate	0.76(0.61–0.96)	0.021	
		Vigorous	0.60(0.50–0.73)	<0.001	
Yes	1121	Light	Reference		
		Moderate	0.67(0.43–1.05)	0.084	
		Vigorous	0.47(0.31–0.70)	<0.001	

The model was fully adjusted for covariates. OR: Odds ratio; CI: confidence interval.

### Mediation analysis

**Figure 3.**
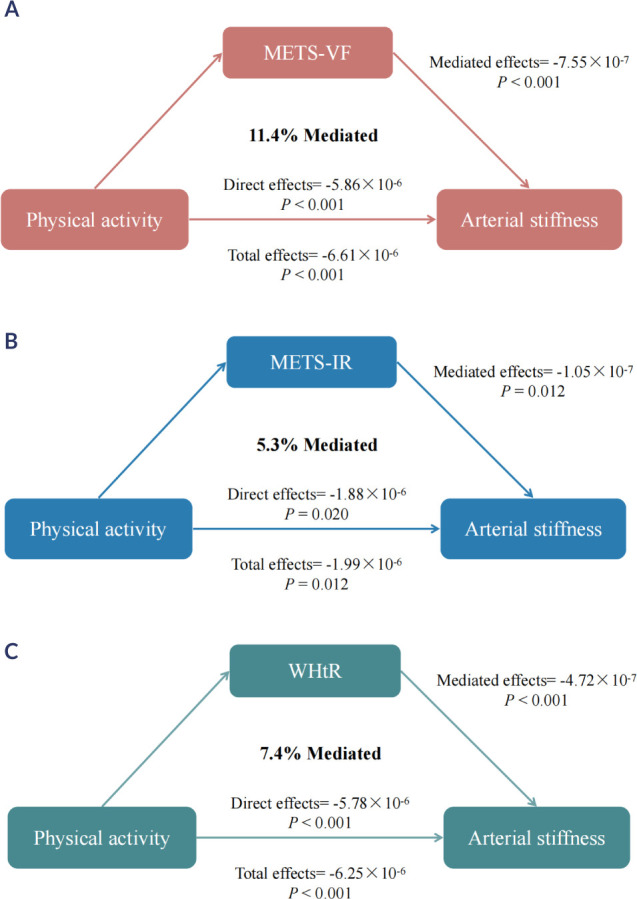
The mediating role of visceral fat metabolism-related indicators in the association between physical activity and arterial stiffness.

To further investigate the biological pathways through which PA affects AS, we systematically evaluated the mediating role of METS-VF and its core components (i.e., METS-IR and WHtR) in the association between PA and AS using the bootstrap method. The mediation analysis (**Figure 3**) revealed that the relationship between PA and AS was significantly mediated by METS-VF, with a mediated proportion of 11.4% (**Figure 3A**). METS-IR and WHtR also acted as notable mediators in the PA-AS relationship, with mediated proportions of 5.3% and 7.4%, respectively (**Figure 3B-C**).

## DISCUSSION

Based on the CHARLS database, this study systematically explored the association between PA and AS risk among middle-aged and older Chinese adults, as well as the potential mediating role of METS-VF within such relationship. A significant negative correlation was identified between PA and AS risk, with more pronounced protective effects observed for moderate-to-high-intensity PA and an overall nonlinear dose-response relationship. No significant interaction effects were observed. Mediation analyses further indicated that METS-VF partially mediated the association between PA and AS, with a mediated proportion of approximately 11.4%. These findings provide novel epidemiological evidence for the biological pathways linking PA to vascular health. Moreover, the results suggested that future vascular health interventions should not only promote regular PA but also consider METS-VF as an auxiliary indicator for risk screening and dynamic monitoring, thereby facilitating early identification of individuals at high risk for AS and providing an objective evaluation of intervention efficacy.

In this study, moderate-to-high-intensity PA was significantly correlated with a lower risk of AS. This result is consistent with findings from the Jackson Heart Study among Black African descent populations, which reported that higher overall PA was independently associated with reduced AS (^32^). The consistent epidemiological evidence across various populations and ethnicities strongly supports the intrinsic link between PA and vascular health. Notably, ePWV used in this study is an estimated indicator calculated based on age, blood pressure, and other parameters, rather than a direct measurement of AS. Previous evidence has established that PA substantially affects blood pressure, a core component of ePWV calculation. Accordingly, the observed association in this study may, in part, reflect PA-induced alterations in blood pressure rather than structural alterations in the arterial wall itself. Such distinction should be fully considered when interpreting ePWV as a surrogate marker of vascular health.

Furthermore, previous studies have reported a similar ceiling effect in the inverse relationship between PA and CVD risk reduction, indicating that further decreases in CVD risk plateau when weekly PA reaches around 3000 MET-min (^33^). Additionally, long-term, high-intensity endurance training has been associated with aortic medial fibrosis, potentially detrimental vascular remodeling, and transient adverse effects on endothelial function (^34^–^36^). In accordance with these prior reports, our RCS analysis revealed a reverse J-shaped curve: protective effects increased rapidly from low to moderate PA levels, then gradually plateaued with further elevations in PA intensity. This dose-response pattern suggests that moderate increases in PA are sufficient to achieve major vascular health benefits, whereas excessive exercise yields limited marginal gain. Nevertheless, the aforementioned vascular risks are primarily derived from observations among elite athletes or those with extreme exercise exposure, which may not be generalizable to routine high-intensity PA among the general population.

This study did not detect any harmful association between high-intensity PA and elevated AS risk, nor did it identify extreme exercise exposure among participants. Therefore, exercise interventions targeting individuals at high risk for AS should follow individualized and progressive principles, prioritizing the establishment of sustainable daily activity habits rather than pursuing excessive high-intensity exercise.

Subgroup analyses showed that high-intensity PA was consistently associated with reduced AS risk across all predefined subgroups, without significant interactions detected. These findings highlight the robustness of the observed association across diverse demographic and baseline health characteristics, providing population-based support among Chinese adults for the concept of “Exercise is Medicine” (^37^). In age-stratified analyses, the inverse correlation between high-intensity PA and AS risk was stronger among individuals aged ≥60 years and older than in those younger than 60. This may be attributed to age-related structural deterioration of blood vessels in older adults, leaving greater room for functional interventions to generate measurable health benefits (^38^–^40^). In analyses stratified by sex, high-intensity PA was linked to a greater reduction in AS risk among females. Existing literature indicates that estrogen may modulate vascular endothelial function, and the female vascular system exhibits higher sensitivity to hemodynamic adjustments after exercise-induced workload changes (^41^–^43^). Moreover, the inverse association between high-intensity PA and AS risk was more pronounced in participants with diabetes, dyslipidemia, or a history of CVD. Such patients typically present with severe endothelial dysfunction and chronic inflammation, both of which are strongly related to increased AS susceptibility (^44^–^47^). Regular PA may ameliorate these pathologies by improving endothelial function and modulating inflammatory responses (^48^,^49^), thereby conferring greater protection in these vulnerable groups.

As a comprehensive indicator integrating metabolic and morphological characteristics, METS-VF may better reflect the pathophysiological activity of VF than single indicators such as body fat percentage or waist circumference (^50^). Our study found that METS-VF mediated 11.4% of the association between PA and AS prevalence. Visceral adipose tissue, as a metabolically active endocrine organ, produces free fatty acids and pro-inflammatory cytokines; excess accumulation leads to systemic chronic inflammation and insulin resistance (^51^,^52^). Such metabolic disturbances are closely implicated in endothelial impairment, vascular smooth muscle cell proliferation, collagen deposition, and ultimately, the development and progression of AS (^53^). Additionally, PA may regulate VF deposition and metabolic homeostasis through multiple biological pathways; notably, PA reduces VF accumulation by elevating energy expenditure (^54^).

Exercise training also induces adaptive adipose tissue remodeling, including enhanced mitochondrial biogenesis and improved mitochondrial function (^55^,^56^), which improve insulin sensitivity and glucose uptake (^57^,^58^). Enhanced mitochondrial oxidative capacity supports lipolytic metabolism and maintains overall metabolic homeostasis (^59^,^60^). Furthermore, PA modulates adipose tissue immune microenvironments and alleviates VF-related chronic inflammation (^61^,^62^). These adaptive changes attenuate the detrimental effects of visceral fat on the vascular system, suggesting that VF burden and metabolic status reflected by METS-VF may serve as a potential intermediate pathway linking PA to AS risk. Nevertheless, despite adjusting statistical models for multiple confounding factors (including BMI), interpreting the observed mediating effects remains complex. Therefore, further validation in prospective cohort or interventional studies is required to clarify the underlying molecular mechanisms.

Despite being based on a nationally representative sample and rigorous mediation analysis, several limitations should be acknowledged. First, the cross-sectional design inherently limits the ability to establish causality. Although mediation analysis suggests plausible physiological pathways, definitive causality among variables require validation through more robust study designs. Second, key variables including PA, AS, and VF were assessed using self-reported data or indirect estimations rather than gold-standard measurements, potentially introducing non-differential misclassification bias and attenuating effect estimates. Third, as the study was based on the CHARLS database, only participants aged ≥45 years were included, which aligns with the core design purpose of this survey. Nevertheless, such age restriction may have limited generalizability of our findings to younger populations. Lastly, diagnostic criteria for dyslipidemia used apply specifically to middle-aged and older Chinese adults aged ≥45 years. Given the discrepancies in lipid reference ranges, ethnic genetic backgrounds, lifestyle patterns, and disease spectrums across regions and countries, caution should be exercised when extrapolating our findings to non-Chinese populations or other age subgroups. Building on our results, future randomized controlled trials in various populations should aim to confirm and elucidate the mechanisms by which exercise may ameliorate AS through VF reduction.

In conclusion, based on the CHARLS database, this study demonstrated that increased PA, particularly at moderate-to-vigorous intensity, is an effective strategy to prevent AS among middle-aged and older Chinese adults. Moreover, the association between PA and AS was found to be partially mediated by VF levels. These findings advance understanding of the mechanisms linking PA to vascular health. From a public health perspective, the results suggest that, alongside promoting regular PA, incorporating VF assessment indicators (e.g., the METS-VF) into health screening systems for middle-aged and elderly populations can provide a reference for identifying individuals at high risk of AS and developing potential exercise-related health intervention strategies.

## Data Availability

the data and materials utilized in the current study are accessible upon reasonable request from the corresponding author.

## SUPPLEMENTARY MATERIAL

**Table S1.** Association between PA and the redefined AS

Variable	Model 1		Model 2		Model 3	
	OR (95%CI)	*p*-value	OR (95%CI)	*p*-value	OR (95%CI)	p-value
Per-SD	0.66 (0.62–0.71)	<0.001	0.69(0.64–0.74)	<0.001	0.71(0.66–0.76)	<0.001
Categorical						
Light	1 (Reference)		1 (Reference)		1 (Reference)	
Moderate	0.75(0.63–0.89)	<0.001	0.85(0.69–1.03)	0.102	0.83(0.68–1.02)	0.075
Vigorous	0.42(0.36–0.49)	<0.001	0.49(0.41–0.58)	<0.001	0.50(0.42–0.60)	<0.001
*p* (trend)		<0.001		<0.001		<0.001

**Model 1** was unadjusted. **Model 2** was adjusted for age, sex, educational level, marital status, and residential area. **Model 3** was further adjusted for age, sex, educational level, marital status, residential area, smoking, alcohol consumption, body mass index, diabetes, dyslipidemia, and cardiovascular disease. OR: Odds ratio; CI: confidence interval.
